# A Vascular Feature Detection and Matching Method Based on Dual-Branch Fusion and Structure Enhancement

**DOI:** 10.3390/s24061880

**Published:** 2024-03-15

**Authors:** Kaiyang Xu, Haibin Wu, Yuji Iwahori, Xiaoyu Yu, Zeyu Hu, Aili Wang

**Affiliations:** 1 Heilongjiang Province Key Laboratory of Laser Spectroscopy Technology and Application, Harbin University of Science and Technology, Harbin 150080, China; 2110603078@stu.hrbust.edu.cn (K.X.); 2220600027@stu.hrbust.edu.cn (Z.H.); aili925@hrbust.edu.cn (A.W.); 2 Computer Science, Chubu University, Kasugai 487-8501, Japan; iwahori@isc.chubu.ac.jp; 3 College of Electron and Information, University of Electronic Science and Technology of China, Zhongshan Institute, Zhongshan 528402, China; yuxy@zsc.edu.cn

**Keywords:** internal cavity features, vascular structure enhancement, vascular feature detection, MIS images, Gaussian weighted fusion, self-adaptive threshold

## Abstract

How to obtain internal cavity features and perform image matching is a great challenge for laparoscopic 3D reconstruction. This paper proposes a method for detecting and associating vascular features based on dual-branch weighted fusion vascular structure enhancement. Our proposed method is divided into three stages, including analyzing various types of minimally invasive surgery (MIS) images and designing a universal preprocessing framework to make our method generalized. We propose a Gaussian weighted fusion vascular structure enhancement algorithm using the dual-branch Frangi measure and MFAT (multiscale fractional anisotropic tensor) to address the structural measurement differences and uneven responses between venous vessels and microvessels, providing effective structural information for vascular feature extraction. We extract vascular features through dual-circle detection based on branch point characteristics, and introduce NMS (non-maximum suppression) to reduce feature point redundancy. We also calculate the ZSSD (zero sum of squared differences) and perform feature matching on the neighboring blocks of feature points extracted from the front and back frames. The experimental results show that the proposed method has an average accuracy and repeatability score of 0.7149 and 0.5612 in the Vivo data set, respectively. By evaluating the quantity, repeatability, and accuracy of feature detection, our method has more advantages and robustness than the existing methods.

## 1. Introduction

The implementation of augmented reality technology in minimally invasive surgery (MIS) lies in the real-time acquisition and integration of internal cavity data by laparoscopy, feedback on its pose and depth information, and how to achieve cognitive display correlation between virtual information and real scenes, thereby reducing doctors’ workload, improving spatial awareness, and expanding vision [1,2]. In response to the observation limitations of laparoscopic fixed display devices, it is necessary to use advanced 3D reconstruction technology to achieve a real-time overlay of preoperative or intraoperative 3D virtual images on the basis of 2D imaging display.

The prerequisite for reconstruction technology is accurate feature tracking. However, for narrow, lack of features, specular reflections, and poor lighting conditions in intracavity environments, feature tracking remains highly challenging [3]. Due to the fact that laparoscopy can only obtain two-dimensional information about the characteristics of the inner cavity, and cannot obtain its depth information through a single observation, when the feature is detected again during the movement of the laparoscope, the three-dimensional spatial information is calculated based on the principle of triangulation using the disparity angle of the laparoscope at different positions. Therefore, how to obtain internal cavity features and perform data matching is a great challenge for laparoscopic 3D reconstruction [4].

Existing research indicates that there are issues in directly applying commonly used visual techniques to MIS due to the involvement of large-scale, free-form tissue deformation and constantly changing surgical scenes that are influenced by complex factors [5]. In this case, the accuracy and efficiency of tracking largely depend on the detection of visual features, which need to exhibit high repeatability under image transformation and robustness under scene changes. The inner cavity environment is very complex, with uneven lighting, high specular reflection, soft tissue deformation in the inner cavity, and often without strong edge features, which pose higher requirements for the robustness of visual processing algorithms to improve the number, repeatability, and distribution of extracted feature points in the medical image processing field.

Vascular features are the most obvious features in the lumen environment, and effective vascular structure enhancement methods can provide useful structural information for later vascular feature extraction. Although the vascular structure is complex and detailed, it shares the common characteristics of a tubular-like structure. The ideal enhancement method should be able to achieve a high and uniform response to the following factors: variable vascular morphology; intensity nonuniformity caused by blood contrast or background; other unrelated structural tissues surrounding blood vessels leading to blurred boundaries; and a large amount of complex information such as background noise [6].

Most of the curve structure enhancement methods are based on the classical Hessian matrix analysis of images, and the research of Frangi et al. has been widely recognized [7]. The method proposed by Meijering et al. further developed the use of Hessian matrices [8]. A Hessian matrix is based on second-order Gaussian derivatives and can be calculated at different scales to enhance features similar to curves, which helps distinguish specific shapes such as circular, tubular, and planar structures. In images, when dark curve structures appear relative to the background, chromaticity measurement is used to describe the image. However, the main drawback of the Hessian matrix is its sensitivity to noise [9]. Due to the large eigenvalues, the curved feature response at the connections is very small, which results in poor performance in dealing with different curved branch structures. In the field of cardiovascular structure enhancement, Huang established an improved non-local Hessian-based weighted filter (NLHWF) to suppress nonvascular structures and noise around real vascular structures in contrast images [10]. In the field of neural structure enhancement, the use of diffusion tensors has a more positive effect on vascular structure enhancement. Jerman et al. used spherical diffusion tensors to overcome the shortcomings of using Hessian matrices, but did not fully solve the problem of low intersection strength [11]. The fractional anisotropic tensor (FAT) measures the variance of eigenvalues between different structures and can detect changes in vascular anisotropy [12]. This feature enables FAT to more reliably enhance the vascular structure, ultimately resulting in a more uniform response. Alhasson proposed a new enhancement function based on FAT, which robustly maintains the relationship between isotropy and anisotropy by considering the degree of anisotropy of the target structure, in order to maintain low amplitude feature values, and successfully achieves high-quality curve structure enhancement for retinal images [13]. Dash et al. proposed a fusion method to enhance the fusion of coarse and fine blood vessels, combined with the advantage of the Jerman filter’s effective response to blood vessel branches, using mean-C threshold segmentation to improve the performance of traditional curved transformation and improve the algorithm speed [14]. The above vascular structure enhancement methods are mainly used for extracting neural structures, coronary angiography, or curve structures of retinal blood vessels, and have not yet been applied in the lumen environment.

Human luminal vascular feature extraction refers to the automatic or semi-automatic extraction of key points of blood vessels from medical images, such as bifurcation points, endpoints, and intersections. The lumen environment measured by laparoscopy is often unknown. Considering factors such as image quality, lighting conditions, background generated by image correction, uncertain environmental descriptions, and noise, robust features can provide effective and accurate two-dimensional information for three-dimensional reconstruction of the lumen environment. The traditional feature extraction methods started with Harris’s definition of corners [15], and then classic methods such as the features from the accelerated segment test (FAST) [16] and scale invariant feature transform (SIFT) [17] were successively proposed. Improved algorithms for SIFT, such as the speed up robust features (SURF) algorithm [18], have better computational speed but poor stability.

Although the oriented FAST and rotated BRIEF (ORB) algorithm [19] solves the rotational invariance in binary robust independent elementary features (BRIEF), it is prone to errors caused by local homogenization of feature points and cumulative drift. Faced with the complex environment of minimally invasive surgery, traditional feature extraction methods applied in large-scale environments with sufficient light and strong edge features are no longer applicable. Therefore, Stamatia proposed an affine invariant anisotropic feature detector based on automatic feature detection (AFD), which only responds to features with low anisotropy, compensating for the shortcomings of LoG and DoG and effectively processing isotropic features [20]. Lin proposed the vessel branching circle test (VBCT) based on Frangi and verified its robustness and saliency for MIS images through monotonic mapping [21]. Subsequently, due to the presence of a certain pixel width in blood vessels, more than one branch point may be detected on a branch, leading to redundancy. Udayakumar put forward the ridgeness-based circle test (RBCT), which simplifies the structure of blood vessels into single pixels, making feature extraction of blood vessels more efficient [22]. They used the patch search process of parallel tracking and mapping (PTAM) [23] to perform feature matching by calculating the zero mean zero sum of squared differences (ZSSD). However, due to the large-scale nature of branch points, their positioning error is about 1.6 ± 0.7 pixels, and the large error introduces uncertainty to subsequent 3D reconstruction. Li et al. added a decision tree based on the C4.5 algorithm to the traditional FAST algorithm, making the feature extraction performance of MIS images more stable and feature point extraction more efficient [24].

On the other hand, rough feature matching methods often have mismatches and the feature point matching methods based on certain structures are adopted according to different application scenarios. For example, matching based on lines and matching based on various stable geometric structures [25]. Davison proposed an active matching method [26], while random sample consensus (RANSAC) and its improved algorithm eliminated the occurrence of mismatches in active matching [27]. Wu propose a feature point mismatch removal method that combines optical flow, descriptor, and RANSAC, to eliminate incorrect feature point matches layer by layer through these constraints and exhibits robustness and accuracy under various interferences such as lighting changes, image blurring, and unclear textures [28]. Liu et al. proposed an improved ORB feature matching algorithm that utilizes non-maximum suppression (NMS) and retinal sampling models to solve mismatch problems in complex environments [29].

In addition to the direct impact of the accuracy of intracavity feature matching on the localization estimation of laparoscopy, the computational complexity of intracavity feature matching is also an important factor affecting the real-time performance of the entire algorithm. Puerto Souza proposed new hierarchical multiaffine (HMA) and adaptive multiaffine (AMA) algorithms to improve the feature matching performance of endoscopic images [30,31]. Zhu proposed a feature matching method for endoscopic images based on motion consensus. A spatial motion field was constructed based on candidate feature matching, and the true match was identified by checking the difference between feature motion and the estimated field, achieving a 3.7 pixel average reprojection error [32]. Li designed a global and local point-by-point matching algorithm based on feature fusion for extracting contour features from gastrointestinal endoscopic images, which used fast Fourier transform (FFT) to reduce the dimensionality of dense feature descriptors and achieve robust and accurate contour feature extraction and matching [33]. Currently, in the medical field, most visual-based 3D reconstruction methods still rely on matching features obtained by traditional methods; even by directly estimating and fusing keyframe depth maps [34] or combining them with camera pose estimation [35] to avoid the need for feature matching. Therefore, feature extraction and matching of the inner cavity environment remains a challenging task.

The main contributions of the proposed method are summarized as follows:

1. To solve the problems of noise and false detection caused by different inner cavity environment boundary areas and highlight areas, we analyze the characteristics of the inner cavity environment, design a targeted preprocessing framework, use a self-adaptive threshold to generate binary masks, introduce a local variance threshold to automatically detect highlight areas, and use the fast marching method (FMM) method for highlight repair.
2. In order to robustly extract effective structural information for vascular features, a Gaussian weighted fusion algorithm for single-pixel enhancement of the vascular structure using dual-branch Frangi measure and multiscale fractional anisotropic tensor (MFAT) is designed. The structural similarity (SSIM) index is introduced to achieve self-adaptive Gaussian weighted fusion, solving the problems of structural measurement differences and uneven response between venous vessels and microvessels, ensuring the continuity and integrity of the vascular structure.
3. To enhance adaptability to different scale vascular structures, a dual-circumference detection of vascular features is implemented. Redundancy is reduced by introducing NMS. Feature matching of neighboring blocks of the results before and after frames is achieved by calculating the zero sum of squared differences (ZSSD).

## 2. Methods

**Figure 1:**
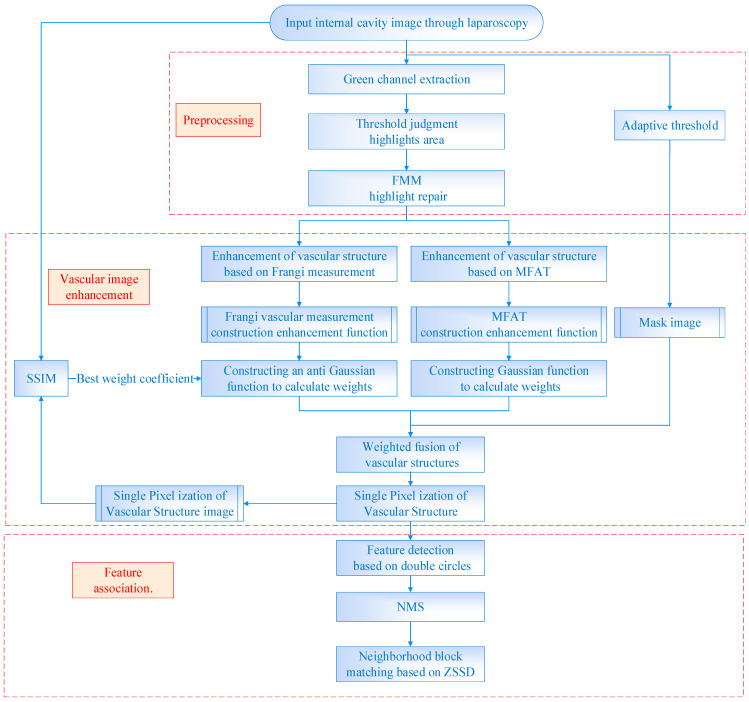
The flowchart of the proposed method.

This paper proposes a method for detecting and matching vascular features based on dual-branch weighted fusion vascular structure enhancement, as shown in Figure 1. An image preprocessing framework is designed based on the characteristics of the internal cavity environment to improve the method’s performance. Binary mask images of boundary regions are generated using a self-adaptive threshold to preserve pixels with valid information in the field of view. The green channel is selected with the highest contrast and the threshold judgment and FMM are used to repair the highlighted areas. Based on the characteristics of the vascular structure, a single-pixel, dual-branch weighted fusion vascular image enhancement algorithm is designed by combining the vascular structure enhancement function based on Frangi measurement and the vascular structure enhancement function based on MFAT. Finally, the double circles detection based on the characteristics of branch points is performed and NMS to extract feature points is introduced. Then, the ZSSD is calculated for the neighboring blocks of feature points extracted from the frames before and after, followed by feature point matching.

### 2.1. Preprocessing

**Figure 2:**
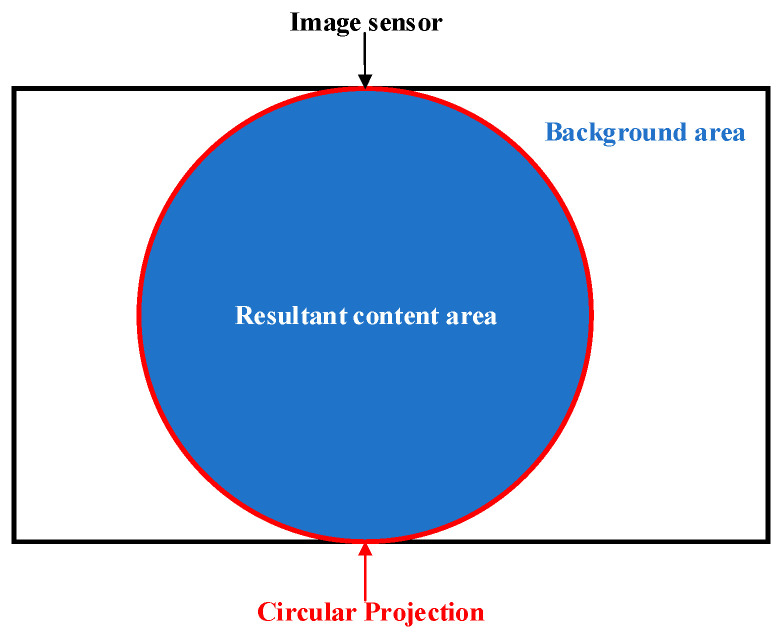
Laparoscopic image sensor area allocation.

As medical optical equipment, endoscopes have a wide variety of types and are applied in different fields. The requirement for minimally invasive surgery limits the size of the optical field that doctors can see. Therefore, in order to maximize the doctor’s field of view, a circular projection created by some laparoscopic descending optical systems is placed inside the image sensor, as shown in Figure 2. As a result, the obtained endoscopic images have noncontent areas, namely dark and uninformed boundary areas, with clear and sharp edges between them and the content areas [36]. For binocular laparoscopy, parallax correction can also result in boundary areas containing noise in the corrected image. At the same time, the large area of bleeding also lacks vascular information, so this paper defines it as a boundary area. The boundary region will generate interference information during image enhancement, so self-adaptive threshold [37] is used to generate effective mask images for different types of laparoscopic images, improving the generalization of the method.

For each pixel (*x*, *y*), the window size centered on it is (*w*, *h*), and the grayscale mean and median of all pixels in the window are calculated to obtain a binary threshold. As shown in Equation (1), pixels (*x*, *y*) that are greater than the threshold are assigned to the foreground or content area, otherwise they are the background or boundary area to obtain a masked image.

(1) $$\mathrm{mask}\left(x,y\right)=\left\{\begin{array}{cc} 1 & I_{m}(x,y)>k\left(\frac{1}{w*h}\right)\sum I\left(x,y\right)-(1-k)\cdot \mathrm{median}(I\left(x,y\right)),\\ 0 & otherwise \end{array}\right.$$

**Figure 3:**
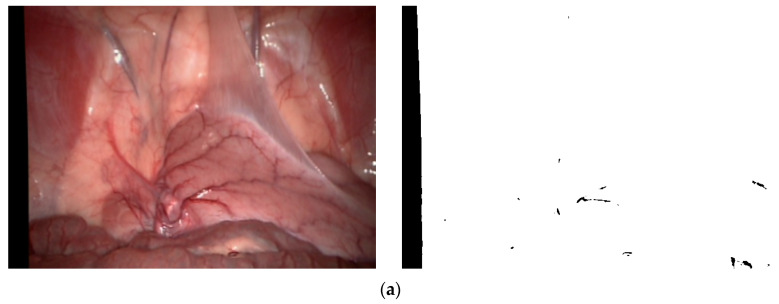

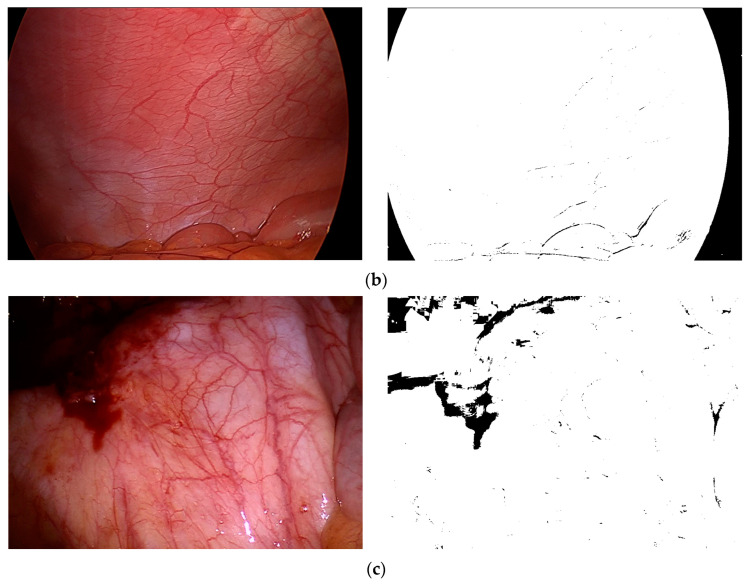
Illustration of derived binary masks of images: (**a**) Rectified01(Hamlyn data set); (**b**) clip2 (Vivo data set); (**c**) clip3 (Vivo data set).

Among them, *I_m_*(*x*, *y*) represents the binarization threshold of pixel points (*x*, *y*); $\left(\frac{1}{w*h}\right)\sum I\left(x,y\right)$ represents the average grayscale around a pixel (*x*, *y*); median(*x*, *y*) represents the median grayscale around a pixel (*x*, *y*); and *k* is the sensitivity coefficient used to control the threshold size. This binary mask image is applicable to various types of MIS images. We selected three typical environments from the Hamlyn data set and Vivo data set, as shown in Figure 3, where (a) represents the left image “0000000000” after inspection and correction in the Hamlyn data set Rectificed01 (binocular) and its corresponding binary mask images, (b) represents the image “base” in the Vivo data set clip2 and its corresponding binary mask images, and (c) represents the image “base” in the Vivo data set clip3 and its corresponding binary mask images.

Among the three RGB channels in MIS images, the most prominent colors for blood vessels are red and green, while the background color is closer to blue. The contrast between the red and blue channels is very poor and noisy, and the corresponding channel pixels do not provide much information about blood vessels [38]. Therefore, the green channel provides the best contrast between blood vessels and the background, with blood vessels being the clearest.

**Figure 4:**
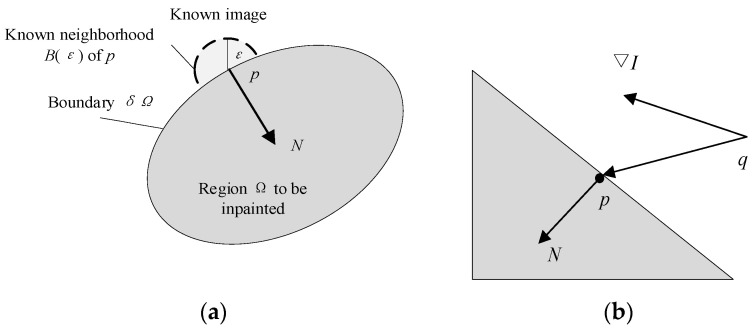
FMM schematic diagram: (**a**) repair area; (**b**) gradient direction.

Using the local variance threshold to automatically identify the highlight areas in the image, based on the special grayscale distribution and spatial distribution characteristics of the highlight areas, the threshold for each pixel point in the image is determined by calculating the variance of the surrounding pixels as follows:

(2) $$T\left(x,y\right)=k\cdot \mathrm{var}\left(R\left(x,y\right)\right)+C$$

where *T*(*x*, *y*) represents the threshold of the local neighborhood centered on (*x*, *y*), *var*(*R*(*x*, y)) represents the pixel value variance of the local neighborhood centered on (*x*, *y*), and *k* and *C* are scaling factors and constants, respectively, used to adjust the size and position of the threshold. Then, FMM is used to achieve highlight restoration, as shown in Figure 4. 

The highlight area is used as the initial boundary condition, and new pixel values are calculated with neighboring points based on the weight of different distances to replace the values of adjacent point sets in the highlight area [39] as follows:

(3) $$I_{q}(p)=I(q)+\nabla I(q)(p-q).$$

Among them, *q* is a point of $B_{\varepsilon }(p)$. Due to the different influence of pixels with different distances within the neighborhood on the new pixel values of point *p*, it is necessary to set the respective weight for each pixel as follows:

(4) $$I(p)=\frac{\sum_{q\in B_{\varepsilon }(p)}w(p,q)\left[I(q)+\nabla I(q)(p-q)\right]}{\sum_{q\in B_{\varepsilon }(p)}w(p,q)}.$$

(5) w(p,q)=dir(p,q)⋅dst(p,q)⋅lev(p,q)

where *w*(*p*, *q*) is the weight function used to quantify the impact of each pixel in the neighborhood on new pixel values. The weight functions are shown in Equations (6)–(8)

(6) $$\mathrm{dir}(p,q)=\frac{p-q}{\left\|p-q\right\|}\cdot N(p)$$

(7) $$\mathrm{dst}(p,q)=\frac{{d_{0}}^{2}}{{\left\|p-q\right\|}^{2}}$$

(8) $$\mathrm{lev}(p,q)=\frac{T_{0}}{1+\left|T(p)-Y(q)\right|}$$

**Figure 5:**
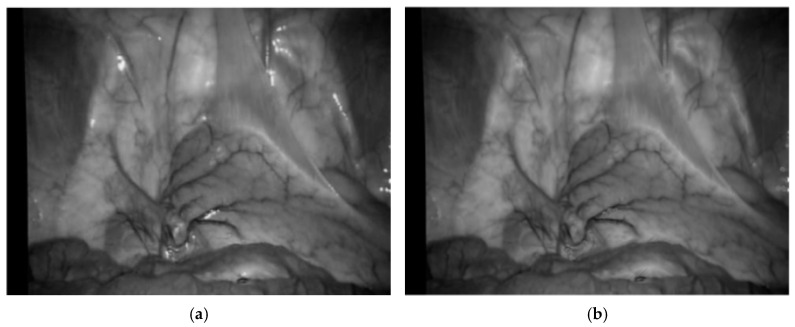
An example of highlight repairing: (**a**) original image; (**b**) result after repairing.

Among them, *d*_0_ and *T*_0_ are distance parameters and horizontal parameters, usually taken as 1. dir(*p*, *q*) is the directional factor, dst(*p*, *q*) is the geometric distance factor, and lev(*p*,*q*) is the horizontal set distance factor. The preprocessing results are shown in Figure 5.

### 2.2. Vascular Structure Enhancement

#### 2.2.1. Analysis of Vascular Features Based on the Hessian Matrix

The Hessian matrix is based on second-order Gaussian derivatives, where the first derivative represents the grayscale gradient and the second derivative represents the rate of change of the grayscale gradient. It can be calculated at different scales to enhance features similar to curves, which helps to distinguish specific shapes, such as circular, tubular, and planar structures. In an image, the chromaticity measurement is used to describe the image when the dark curve structure appears relative to the background. The Hessian matrix *H* is used to calculate the eigenvalues $\lambda_{1}$ and $\lambda_{2}$ pixel by pixel for *L*, which represent the anisotropy of image changes in the two eigenvector directions. By analyzing the symbols and sizes of Hessian eigenvalues, as shown in Table 1, the selective enhancement can be performed on local image structures independent of direction based on the shape of the structure and foreground and background brightness. The spotted structure tends to be isotropic, and the stronger the linearity, the more anisotropic the structure. This method can be used to distinguish the linear vascular structures in the inner cavity, as shown in Equation (9)

(9) $$H=\left(\begin{array}{cc} H_{11} & H_{12}\\ H_{21} & H_{22} \end{array}\right)=\left[\begin{array}{cc} \frac{\partial^{2}L}{\partial x^{2}} & \frac{\partial^{2}L}{\partial x\partial y}\\ \frac{\partial^{2}L}{\partial y\partial x} & \frac{\partial^{2}L}{\partial y^{2}} \end{array}\right]$$

Ideally, in a two-dimensional image, it is assumed that |*λ*_1_| ≤ |*λ*_2_|, where *H* represents high eigenvalues, L represents low eigenvalues, N represents noise, +/− represents the sign of the eigenvalues, and λ_2_ represents a bright (dark) structure on a dark (bright) background. The tubular structure is a vascular target that enhances the human lumen.

#### 2.2.2. Vascular Enhancement Based on the Frangi Measure Filtering

The classic Frangi filter is an enhanced filtering algorithm based on the Hessian matrix [7], where the value *R_B_* is approximately zero when the image structure is a tubular structure; that is, a blood vessel. The Frangi vascular enhancement function uses response *R_B_* to distinguish between speckled and tubular structures, and measures *S* to distinguish the background. The calculation is shown in Equations (10) and (11)

(10) $$R_{B}=\left|\frac{\lambda_{2}}{\lambda_{1}}\right|$$

(11) $$S=\sqrt{{\lambda_{1}}^{2}+{\lambda_{2}}^{2}}$$

Then, construct an enhancement function based on *R_B_* and *S* as shown in Equation (12)

(12) $$F(x,y,\sigma )=\left\{\begin{array}{cc} 0 & \lambda_{1}\geq 0,\\ \exp(\frac{{R_{B}}^{2}}{2\cdot \beta^{2}})\cdot (1-\exp(\frac{-S^{2}}{2\cdot c^{2}})) & \mathrm{otherwise} \end{array}\right.$$

where *β* is used to adjust the sensitivity of the distinguishing block and strip regions, and *c* is used to adjust the overall smoothness of the filtered image.

#### 2.2.3. Vascular Enhancement Based on MFAT

The Frangi vascular enhancement function aims to suppress the components of circular structures, but not all vascular structures elongate. In the process of exploring three-dimensional vascular structure enhancement, image analysis is usually closely related to the formation of neural processes and dihedral differentiation. Jerman et al. analyzed multiple vascular enhancement functions and summarized the advantages and disadvantages of solving vascular structure enhancement problems based on different functions [11]. For two-dimensional images, the Frangi vascular enhancement function was simplified. Based on $\;e^{x}\approx 1+x$, it was found that

(13) $$F\prime (x,y,\sigma )=1-\exp(\frac{-S^{2}}{2\cdot c^{2}})\approx \frac{1}{2c^{2}}\left(\lambda_{1}^{2}+\lambda_{2}^{2}\right)$$

Through analysis, it was found that the main reason for Frangi’s function design, which is proportional to the size of the feature values, is to suppress noise in low-intensity and uniform-intensity image regions, where all feature values have low and similar sizes. This method usually relies on the values of eigenvalues, which leads to several problems as follows: (1) in a slender or circular structure with uniform strength, the eigenvalues are uneven, (2) the feature values vary with the intensity of the image, and (3) enhancement is uneven at different scales. Therefore, redefining characteristic values, −*λ* is for bright structures on a dark background, and *λ* is for dark structures on a bright background.

According to Meijering et al. [8], the Hessian matrix has been improved as shown in Equation (14)

(14) $$H\prime =\left[\begin{array}{cc} H_{11}+\alpha H_{22} & \left(1-\alpha \right)H_{12}\\ \left(1-\alpha \right)H_{12} & H_{22}+\alpha H_{11} \end{array}\right]$$

Then, construct neuron measures as shown in Equations (15) and (16)

(15) $$N=\left\{\begin{array}{cc} \frac{\lambda_{\max}}{\lambda_{\min}} & \mathrm{if}\;\lambda_{\max}<0,\\ 0 & \mathrm{if}\;\lambda_{\min}\geq 0, \end{array}\right.,\;\lambda_{\max}=\max\left(\lambda_{1}{,\;\lambda }_{2}\right),\;\lambda_{\min}=\min\left(\lambda_{\max},\;\lambda_{1}^{\prime },\;\lambda_{2}^{\prime }\right)$$

(16) $$\lambda_{1}^{\prime }=\lambda_{1}+\alpha \lambda_{2},\lambda_{2}^{\prime }=\lambda_{2}+\alpha \lambda_{1}$$

Among them, *λ_max_* is the maximum eigenvalue and *λ_min_* is the minimum eigenvalue, representing the normalized eigenvector and eigenvalues of *H*′. The above neuron measures suppress background intensity discontinuity points and darker linear structures that are immune to first-order derivatives (*λ_max_* (*x*) ≥ 0). Meanwhile, the neuron measure is a scalable function that can be used as a scale parameter *σ* Gaussian kernel and is used to suppress noise. Therefore, Alhasson et al. proposed an improved diffusion tensor measure FAT based on to enhance the vascular structure in three-dimensional images [40]. We applied it to two-dimensional internal cavity images and introduced auxiliary techniques *λ*_3_, which is defined as the three-dimensional shape of blood vessels in a two-dimensional image to obtain the regularized eigenvalues *λ_p_* and its cutoff threshold *τ*. Construction *λ_p_*_1_, *λ_p_*_2_ through the upper limit cutoff threshold is passed *τ_1_* and lower cut-off threshold *τ*_2_*,* respectively. And *λ_p_*_1_, *λ_p_*_2_ are used to adjust *λ*_3_

(17) $$FAT_{\lambda }^{\sigma }=\sqrt{\frac{3}{2}}\sqrt{\frac{{\left(\lambda_{\rho 1}-\bar{D_{\lambda }}\right)}^{2}+{\left(\lambda_{2}-\bar{D_{\lambda }}\right)}^{2}+{\left(\lambda_{\rho 2}-\bar{D_{\lambda }}\right)}^{2}}{\lambda_{\rho 1}^{2}+\lambda_{2}^{2}+\lambda_{\rho 2}^{2}}}$$

(18) $$\bar{D_{\lambda }}=\frac{\lambda_{p1}+\lambda_{2}+\lambda_{p2}}{2},\;\lambda_{p}=\left\{\begin{array}{ccc} \lambda_{3}\left(x,\sigma \right) & \mathrm{if}\;\lambda_{3}\left(x,\sigma \right)>\tau \max_{x}\left(\lambda_{3}\left(x,\sigma \right)\right), & \\ \tau \max_{x}\left(\lambda_{3}\left(x,\sigma \right)\right) & \mathrm{if}\;0<\lambda_{3}\left(x,\sigma \right)\leq \tau \max_{x}\left(\lambda_{3}\left(x,\sigma \right)\right), & \\ 0 & otherwise & \end{array}\right.$$

Correspondingly, to suppress background noise, construct a response $R_{\lambda }^{\sigma }$ as shown in Equation (19)

(19) $$R_{\lambda }^{\sigma }=\left\{\begin{array}{ccc} 0 & \mathrm{if}\;\lambda_{\rho }>\lambda_{\rho }-\lambda_{2}\vee \lambda_{\rho }\geq 0\vee \lambda_{2}\geq 0\vee >\lambda_{\rho }-\lambda_{2}\max_{x}\left(\lambda_{\rho }-\lambda_{2}\right), & \\ 1 & \mathrm{if}\;\lambda_{\rho }-\lambda_{2}=\min_{x}\left(\lambda_{\rho }-\lambda_{2}\right), & \\ 1-{\bar{MFAT}}_{\lambda }^{\sigma } & \mathrm{otherwise} & \end{array}\right.$$

According to the concept of amplitude regularization, construct a multiscale enhancement function ${\bar{MFAT}}_{\lambda }$ based on the maximum cumulative response through each scale *σ*, as shown in Equations (20) and (21)

(20) $${\bar{MFAT}}_{\lambda }^{\sigma }={\bar{MFAT}}_{\lambda }^{\sigma -1}+\sigma tanh\left(R_{\lambda }^{\sigma }-\sigma \right)$$

(21) $${\bar{MFAT}}_{\lambda }=max_{\sigma }\left({\bar{MFAT}}_{\lambda }^{\sigma },R_{\lambda }^{\sigma }\right)$$

#### 2.2.4. Gaussian Weighted Fusion and Single-Pixel Vascular Structure

The Gaussian function has a higher weight near the center, and as the distance from the center increases, the weight gradually decreases. This paper combines two enhancement results by constructing a Gaussian weighting function to achieve compensation. The vascular enhancement result after Gaussian weighted fusion, denoted as *I_vessel_*, is shown in Equation (22)

(22) $$I_{\mathrm{vessel}}=\frac{w_{1}}{w}\cdot I_{\mathrm{Frangi}}+\frac{w_{2}}{w}\cdot I_{\mathrm{MFAT}}$$

Among them,

(23) $$w=w_{1}+w_{2}$$

(24) w1=e−I−IFrangi22⋅μ2,w2=1−e−I−IMFAT22⋅μ2

Among them, *w* is the Gaussian weight; *I_Frangi_* and *I_MFAT_* are the Frangi enhancement results and MFAT enhancement results, respectively; and *μ* is the parameter that controls the shape of the Gaussian function. Considering that there is no gold standard for the structure of the lumen blood vessels, the optimal weights for different types of images are obtained based on SSIM, which refers to the parameter with the best score between the vascular structure and the lumen image, thereby improving the generalization of fused image applications.

**Figure 6:**
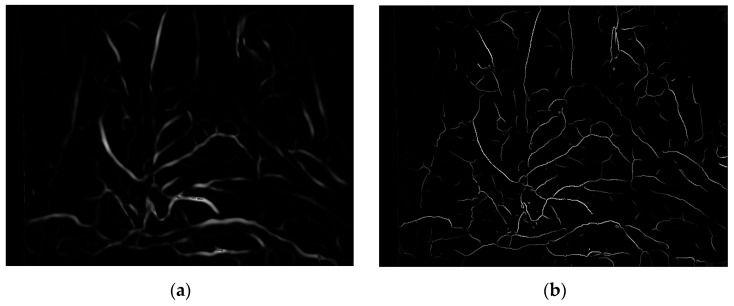
Enhancement results of vascular structure enhancement: (**a**) the Frangi enhancement method; (**b**) the method proposed in this paper.

Blood vessels have a certain width, and the multipixel vascular features used for the vascular structure cannot accurately locate branch points and segments. Therefore, single-pixel vascular centerlines can be used to replace the original vascular morphology. Analyze the grayscale gradient changes of all pixels in the width direction of blood vessels, and use weighted operations to calculate the *I_ridge_* of the vessel centerline, as shown in Equation (25)

(25) $$\begin{array}{l} I_{\mathrm{ridge}}(x,y,\vartheta )=I_{\mathrm{vessel}}(x,y,\vartheta )\\ \cdot \mathrm{abs}\left\{\mathrm{sign}(\nabla I(x+\in u_{2},u+\in v_{2},\varepsilon ))-\mathrm{sign}(\nabla I(x-\in u_{2},u-\in v_{2},\vartheta ))\right\}/2 \end{array}$$

where ∇ is the gradient operator, ${\left(u_{2},\;v_{2}\right)}^{T}=V_{2}$ and *ε* = 1.0 is the pixel width. However, most of the blood vessel width obtained are two pixels. In order to obtain a more accurate single-pixel width ridge, it is necessary to obtain the local maximum value of each centerline pixel in the direction of the feature vector $V_{2}$, as shown in Equation (26)

(26) $$I_{\mathrm{ridge}}(\cdot )=\left\{\begin{array}{cc} I_{\mathrm{ridge}}(\cdot ), & \mathrm{if}\;I_{\mathrm{ridge}}(x\pm \in u_{2},y\pm \in v_{2},\vartheta )<I_{\mathrm{ridge}}(\cdot )\;\\ 0, & \mathrm{otherwise} \end{array}\right.$$

where *I_ridge_* represents ridge degree. As shown in Figure 6, (a) is the enhancement result of the Frangi enhancement method, and (b) is enhancement result of this paper method. The blood vessels after single-pixel transformation are thinner and have clearer branches compared to the original method blood vessel images in the human lumen.

Algorithm 1 describes a Gaussian weighted fusion of dual-branch Frangi measures and MFAT for single-pixel enhancement of vascular structures.
**Algorithm 1**Input: Inner cavity image *I*, preprocessed inner cavity image *I_pre_*, masked image *I_mask_* Output: Single-pixel vascular image *I_ridge_*
1:Branch 1 uses a blood vessel enhancement algorithm based on Frangi measure filtering on *I_pre_*2:***for*** each pixel *p* ϵ *I_pr**e**_* ***do***3:   ***for*** each scale ***σ*** ***do***4:    Calculate the Hessian matrix and its eigenvalues (*λ_1_*, *λ_2_*) and eigenvectors (*I_x_*, *I_y_*)5:    Calculate *Direction*, *R_B_*, and ***S*** based on eigenvalues and eigenvectors6:    At scale ***σ*** Calculate the Frangi measure filtering result *F*(*σ*)7:   ***end for***8:   Extract the maximum response and corresponding filtering result *max*(*F*(*σ*))9:***end for***10:Combining ***I_mask_*** to generate Frangi measure vascular enhancement images *I_Frangi_*, *Direction*11:Branch 2 using MFAT based vascular enhancement on *I_pre_*12:***for*** each pixel *p* ϵ *I_pr**e**_* ***do***13:   ***for*** each scale ***σ do***14:    Calculate the Hessian matrix and its eigenvalues (*λ*_1_,*λ*_2_) and *Direction*15:    Set λ_3_ = λ_2_. Construct new eigenvalues based on the eigenvalues λ_*p*1_, λ_*p*2_, and calculate $FAT_{\lambda }^{\sigma },\;R_{\lambda }^{\sigma }$16:    At scale σ calculate and update MFAT results ${\bar{MFAT}}_{\lambda }$17:   ***end for***18:   Remove pixels ${\bar{MFAT}}_{\lambda }$ < 1 × 10^−2^19:   Extract the maximum response and corresponding scale filtering result *max*$({\bar{MFAT}}_{\lambda }$(*σ*))20:***end for***21:***for*** every steps *μ*ϵ (2, 4) ***do***22:   Establish a Gaussian model and calculate weights *w_1_* and *w_2_*23:   Weighted fusion of *I_Frangi_* and *I_MFAT_* to generate binary image *I_vessel_*24:   Using single-pixel algorithm based on *I*, *I_vessel_*, and *Direction*25:   Gaussian Filter Smoothing Image *I*26:   Generate grid coordinate matrix [*X*, *Y*]27:   ***for*** each coordinate (*x*, *y*) ϵ [*X*, *Y*] ***do***28:    Calculate the feature direction gradient ∇ and inner product29:    If there is a sign change in the gradient along the direction of maximum curvature, it is considered as a vascular ridge line *mask_ridge*30:    Multiply the ridge mask with the vascular image to obtain the vascular centerline image *I_ridege_*31:     *if* the single-pixel detection flag for blood vessels is true32:     Interpolate and shift vascular images to obtain *I_Rshift1_* and *I_Rshift2_*33:     Compare to obtain a single-pixel ridge *mask_ridge2* and its application34:     ***end if***35:    ***end for***36:   Generate a single-pixel vascular image *I_ridge_*37:   Calculate the SSIM score between *I* and *I_ridge_*38: ***end for***39:Select the corresponding optimal parameter *μ* based on the maximum SSIM score40:Calculate the optimal weights *w_1_* and *w_2_*41:Weighted fusion of *I_Frangi_* and *I_MFAT_* to generate binary image *I_vessel_*42:Generate a single-pixel vascular image *I_Frangi_* using a single-pixel algorithm based on *I*, *I_vessel_*, and *Direction*

### 2.3. Circle Detection of Vascular Branches

**Figure 7:**
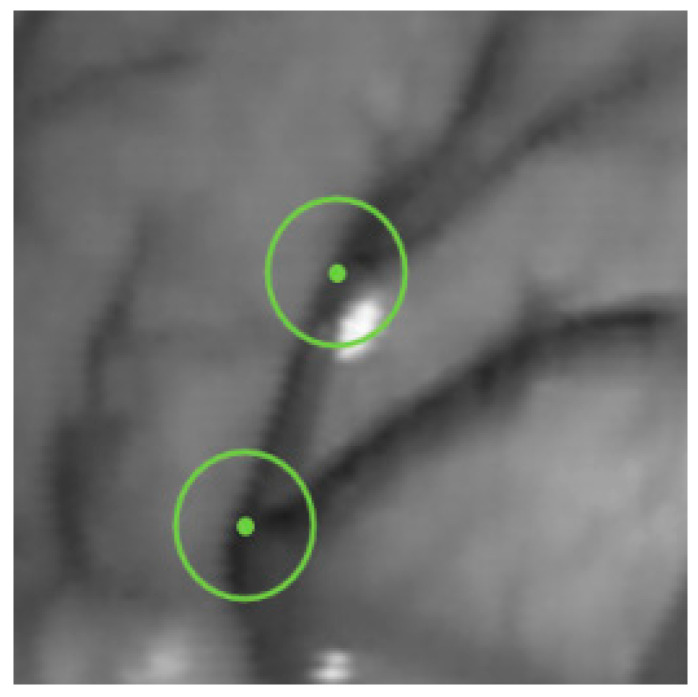
Vascular branching points.

A vascular branch point is a feature point with three or more vascular branch segments centered on it. Due to rotation and scaling, the vascular branch points can still be detected in intracavitary vessels with a single texture feature and strong robustness. Based on this definition and the principles of the FAST algorithm, a single-pixel wide blood vessel image that has undergone image preprocessing is defined as a blood vessel branch point when there are three or more identical pixel values between the center candidate point and the point on the circumference. This method is called the circle detection of vascular branches (CDVB), and the intersection of the circumference with three or more blood vessels will cause a black and white change in the color of the pixels on the circumference, as shown in Figure 7.

Although blood vessels have been pixelated, there is still a possibility of multiple pixels at the intersection of blood vessels and circles in the CDVB algorithm. In order to determine the accurate position of the intersection pixels, all pixel values along the circumference are counted, and the peak coordinates are taken as the intersection coordinates. Set a threshold to determine feature points, as shown in Equation (27)

(27) $$N=\sum_{x\forall (circle(p))}\left|I_{x}-I_{p}\right|>\varepsilon_{d}$$

where *P* is a candidate detection target point with a pixel value of *I_P_*, and *r* is the radius of the circle. Set a threshold $\varepsilon_{d}$, and subtract the pixel values on the circle from the pixel values at point *P* to obtain *I_P_* − *I_x_*. Compare this value with threshold $\varepsilon_{d}$. If there are *N* consecutive pixels with a difference greater than $\varepsilon_{d}$ from *I_P_*, then the candidate point will be judged as a feature point.

In order to enhance the recognition ability of microvascular branching points, reduce false detections, and enhance algorithm adaptability and flexibility, set different radius double circles on the candidate points for judgment. If one of the circles is successfully judged, it will be marked as a feature point and its coordinates and peak points will be recorded.

This method can accurately detect the branching points of blood vessels in the human lumen. However, there still exists uneven distribution of feature points, especially the accumulation of local feature points. There are two reasons for this type of accumulation: first, there are multiple effective corner points in a small area, and second, due to the width of blood vessels, it is highly possible to detect multiple feature points in a single effective corner area, with all but one being redundant. This situation not only increases the computational burden, but also poses a risk of reducing the accuracy of feature point matching.

For the above situations, NMS is introduced to solve the problem of uneven distribution of feature points [41]. Perform connectivity analysis based on the peak point of feature point *P* in the neighborhood, and obtain the score value of the score function *V* for the connected component calculation. If *P* is the point with the highest score value in the neighborhood, it is kept:

(28) $$V=\max\left\{\begin{array}{cc} \sum (I_{T}-I_{P})-\varepsilon_{d}\; & {\mathrm{if}\;I}_{T}\in b,\\ \sum (I_{P}-I_{T})-\varepsilon_{d}\; & {\mathrm{if}\;I}_{T}\in d \end{array}\right.$$

where *V* represents the score, *I_T_* represents the pixel value of the template point, *b* is the bright spot, and *d* is the dark spot.

### 2.4. Neighborhood Block Branch Point Matching Based on ZSSD

Based on the feature point neighborhood block matching algorithm in PTAM, the sum of squared errors is calculated within the neighborhood range of the matching points, and the matching point pairs are determined by the value of the sum of squared errors. In the prematching graph S, take a small rectangular graph in S with an area of *M* × *N*. The starting coordinate of the rectangular graph is the coordinate of the upper left corner (*i*, *j*). The template that matches the small rectangular graph is found through the traversal method. Whether it matches or not needs to be determined by calculating the value of the sum of squared errors as follows:

(29) $$D(i,j)=\sum_{s=1}^{M}\sum_{t=1}^{N}[S(i+s-1,j+t-1)-T(s,t){]}^{2}$$

Among them *S*(*x*, *y*) is the search image of size *M* × *N*, and *T*(*x*, *y*) is the template image of *M* × *N*. 1≤i≤*m* − *M* + 1, 1≤J≤*n* − *N* + 1. The smaller the average absolute difference *D*(*i*, *j*), the more similar it is. Therefore, just find the smallest *D*(*i*, *j*) to determine the position that can match the block.

Based on the block search process, the information from previous frames is first saved by defining a search area for each map point in the current frame. For each *P* point saved in the frame, its corresponding point *Q* in the current frame can be obtained through homography mapping. The search area of *P* is a circular area centered on *Q*, with a radius of 1/20 of the image width. Second, select the feature points located within the neighborhood in the current frame, which are called neighboring points. Compare the 21 × 21-sized neighborhood blocks of each neighboring point with the same-sized neighborhood blocks of point *P*. Apply the affine warping obtained from the ground truth homography mapping to the local blocks of point *P* to accommodate changes in viewpoints, and calculate the ZSSD for each block. Finally, if the ZSSD value of a neighboring point is the minimum and is less than the predefined threshold of 0.02, it is considered to be matched. The condition for determining the correct pair of corresponding points is $\left|Q^{\prime }-Q\right|<$ 3.5 pixel.

## 3. Results

In order to demonstrate the superiority of the algorithm proposed in this paper, the Vivo data sets were used, including abdominal wall and small flat tissue surfaces in the anterior part of the pelvis captured during laparoscopic colon surgery. The data set collection range allowed for global homology mapping, including a base image for matching with other images, manually labeled 20 well distributed feature point pairs, and ground truth homogeneous mapping. The experimental environment is Matlab R2019a, the running environment is Windows 10, and the computer configuration is Intel Core i5-5200U CPU @ 2.20 GHz. The computer brand is Asus, and the origin is Suzhou, China.

### 3.1. Weighted Coefficient Analysis of Vascular Structure Enhancement

**Figure 8:**
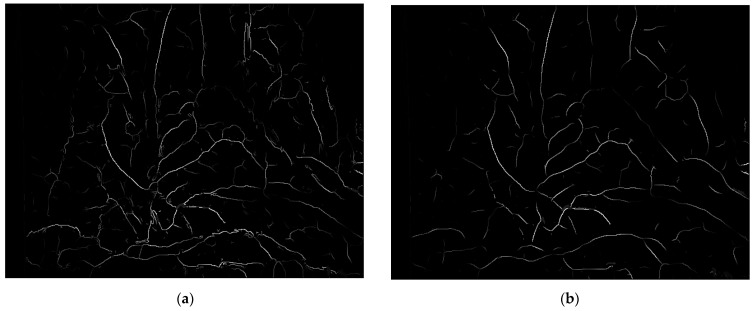
Weight parameter analysis: (**a**) *μ* = 1; (**b**) *μ* = 5.

The analysis was conducted from both subjective and objective perspectives. When the weight was too small, there was severe distortion in the visual effect of single-pixel vascular structures. When the weight was too large, there was a significant loss of fine blood vessels. Experiments were conducted for *μ* = 1 and *μ* = 5, as shown in Figure 8.

**Figure 9:**
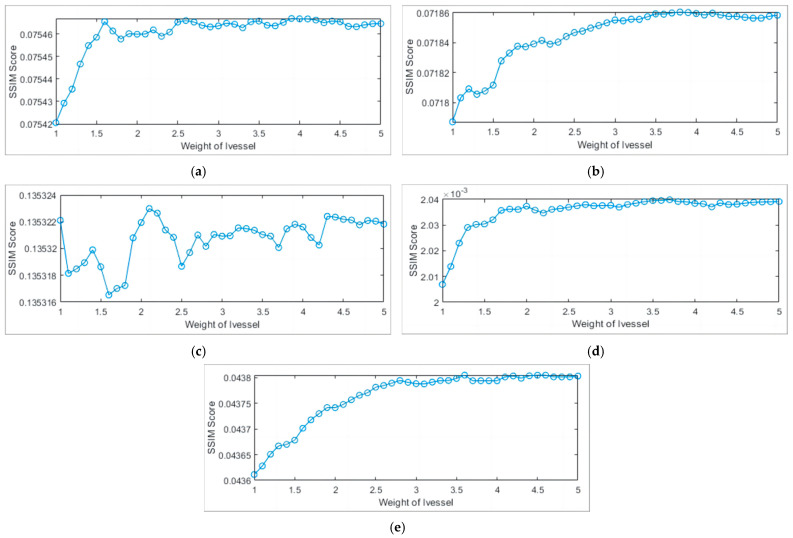
SSIM analysis: (**a**) clip1; (**b**) clip2; (**c**) clip3; (**d**) clip4; (**e**) clip5.

SSIM is used as an analytical indicator to reflect the similarity between the original image and the enhanced vascular structure image, as shown in Equation (30)

(30) $$SSIM(I,I_{ridge})=\frac{\left(2\times \mu_{I}\times \mu_{I_{ridge}}+C_{1})(2\times \delta_{II_{ridge}}+C_{2}\right)}{\left(\mu_{I}^{2}\times \mu_{I_{ridge}}^{2}+C_{1})(\delta_{I}^{2}\times \delta_{I_{ridge}}^{2}+C_{2}\right)}$$

where *I*, *I_ridge_* represents the inner cavity image and single-pixel vascular image, $\mu_{I}$ and $\mu_{I_{ridge}}$ is the average pixel value of *I* and *I_ridge_*, $\delta_{I}^{2}$ and $\delta_{I_{ridge}}^{2}$ is the variance of pixel values for *I* and *I_ridge_*, and $\delta_{II_{ridge}}^{\;}$ is the covariance of pixel values for *I* and *I_ridge_*. *C*_1_ and *C*_2_ are constants, ensuring that the denominator is not zero. As shown in Figure 9, although the significant difference in visual effects between the two results in low SSIM scores, the limited vascular structure information can help us evaluate the similarity between the fusion results and the original image, in order to better understand the quality of the fusion results and make necessary optimizations. This paper introduces the SSIM index to automatically obtain the current optimal parameters for Gaussian weighted fusion of images, improving the accuracy and reliability of fusion results. The highest SSIM scores for the five data sets are shown in the Table 2, and the optimal parameters are concentrated between 2 and 4. Therefore, in order to improve the algorithm speed, the parameter range is set to (2, 4).

### 3.2. Analysis of Feature Points Extraction and Matching

**Figure 10:**
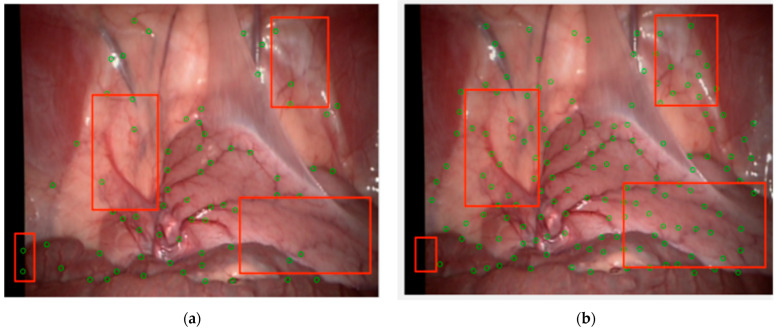
Extraction of vascular branch points: (**a**) after Frangi enhancement; (**b**) after weighted fusion MFAT enhancement.

Subjectively, taking images from the Hamlyn data set Rectificed01 as an example, on the basis of enhancing the vascular structure, circles with a radius of 5 and 7 are respectively set for detection and extraction of branch points. Compared with the classical Frangi enhancement algorithm, the results are shown in Figure 10 and the green circles marked the feature points. It can be seen that the method proposed in this paper compensated for the structural measurement differences and uneven responses of the Frangi enhancement method on venous blood vessels and microvessels, suppressed the influence of boundary regions and highlights, solved the problem of generating a uniform response between different vascular structures, and effectively suppressed noise without affecting the connection as shown the regions in the red rectangles. The method proposed in this paper covers the preprocessing framework and the fusion optimization process. Although vascular enhancement is achieved through effective parallel processing, it still affects the overall running speed of the method. The average time of the RBCT is 0.95931 s, while the method in this paper increases it to 1.9515 s.

**Figure 11:**
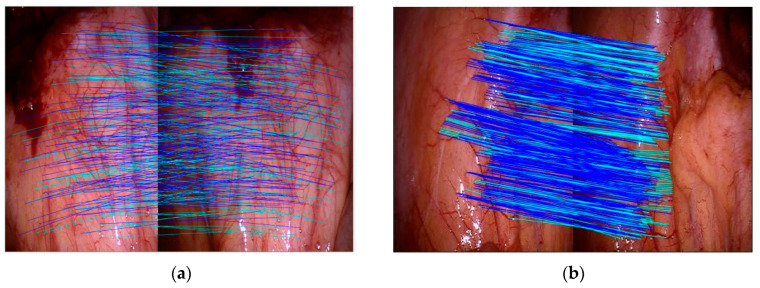
Illustration of matching vascular branching points: (**a**) clip3 (Vivo data set); (**b**) clip4 (Vivo data set).

Objectively speaking, based on the feature point neighborhood block matching algorithm in PTAM, ZSSD is calculated for feature matching Experiments are conducted on base images from five data sets and a random image. For a comprehensive evaluation, the number of branch point extracts, repeatability, average error, and variance are used as evaluation indicators. Repeatability is the percentage of points detected in images from different viewpoints. The average error is the average mean of all matching points. Variance represents the distribution of feature points, and the larger the variance, the wider the distribution range. The results are shown in Table 3 compared with the method proposed in this paper and RBCT and the adaptive FAST (AFAST) algorithm by Liu et al. [42]. The matching effect is shown in clip3 and clip4 as examples, as shown in Figure 11. The above results indicate that the method proposed in this paper has significantly improved the number of extracted branch points, repeatability, and variance compared to the original method, and the average error has generally decreased.

## 4. Discussions

The prerequisite for reconstruction technology is accurate feature tracking, which is a well-researched topic in computer vision. Existing research indicates that there are significant issues in directly applying commonly used visual techniques to MIS due to the involvement of large-scale, free-form tissue deformation and constantly changing surgical scenes that are influenced by complex factors. In this case, the accuracy and efficiency of tracking largely depend on the detection of visual features, which need to exhibit high repeatability under image transformation and robustness under scene changes. This paper analyzes the differences between MIS images and images of the retina, neurons, etc., in response to the lack of robust and uniformly distributed features in the inner cavity space. For MIS images containing rich specular reflections, uneven lighting areas, smoke, and other factors, further research and design were conducted on a method for detecting and associating vascular features based on dual-branch weighted fusion vascular structure enhancement.

The preprocessing process analyzes the factors that lead to incorrect extraction of branch points in different internal cavity environments. The self-adaptive threshold is used to generate binary mask images and introduce them into the fusion processing stage. The threshold judgment and FMM method are used for highlight repair to suppress the influence of boundary and highlight regions. The visual effect of the experimental results shows the effectiveness of the method proposed in this paper.

The enhancement process combines the advantages of two enhancement algorithms to design a Gaussian weighted fusion of dual-branch Frangi measure and MFAT for single-pixel enhancement of the vascular structure. The process introduced SSIM indicators as self-adaptive standards to improve algorithm accuracy and reliability, analyzed indicators to determine the weight parameter range applicable (2, 4) to the inner cavity environment, and improved the algorithm speed.

**Figure 12:**
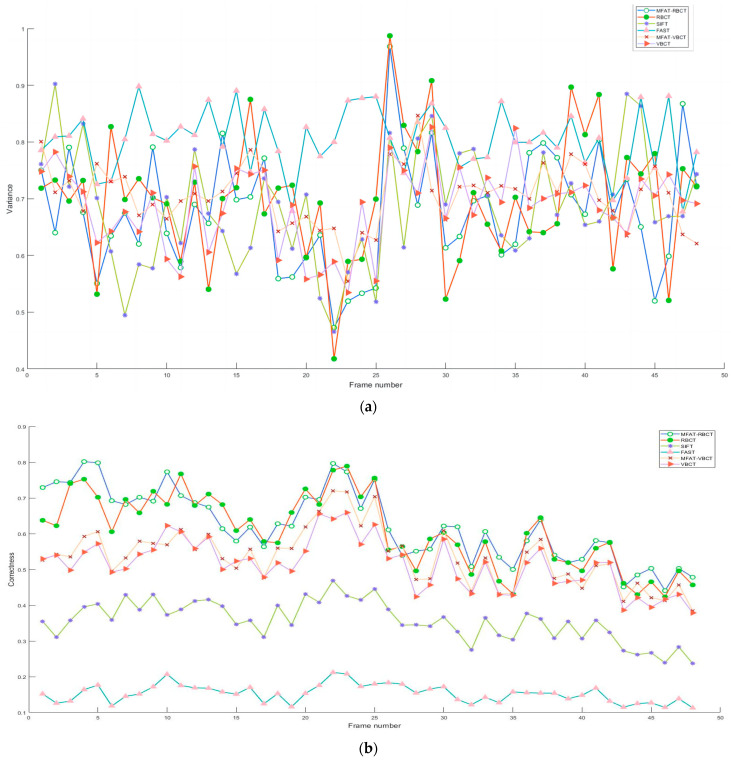

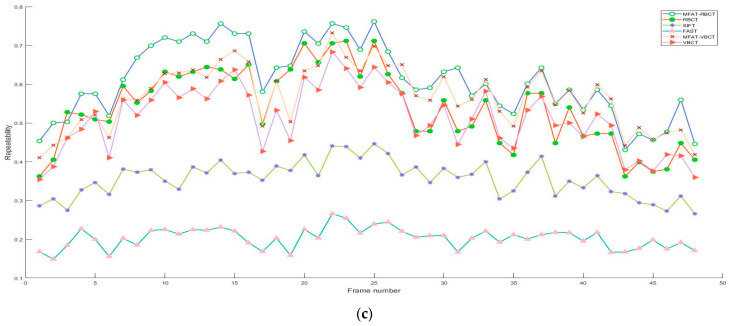
Indicator analysis: (**a**) variance of error of detectors; (**b**) correctness of matching; (**c**) repeatability over viewpoints.

The feature extraction and associating process set up dual-circumference detection combined with the NMS algorithm to extract vascular feature points, and calculated ZSSD for branch point matching based on the feature point neighborhood block matching algorithm. Subjectively, the experimental results indicated that the algorithm proposed in this paper compensated for the structural measurement differences and uneven responses of the Frangi enhancement method to venous vessels and microvessels, providing effective structural information for later vascular feature extraction. Objectively, a detailed analysis was conducted on the methods proposed in this paper (MFAT-RBCT, MFAT-VBCT), and classical algorithms RBCT, SIFT, FAST, and VBCT. VBCT did not perform single-pixel processing on the vascular structure during the processing. The experiment is divided into frame rate analysis for all data sets and overall analysis for a single data set. As shown in Figure 12a, b, c, respectively, this study analyzed the variance, matching accuracy, and repeatability of six methods for all frame rates in data set clip1. The accuracy and repeatability of single-frame matching have been improved compared to RBCT and VBCT, and are significantly better than FAST and SIFT.

**Figure 13:**
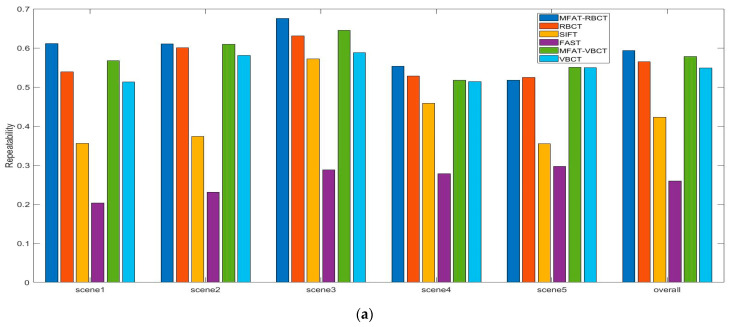

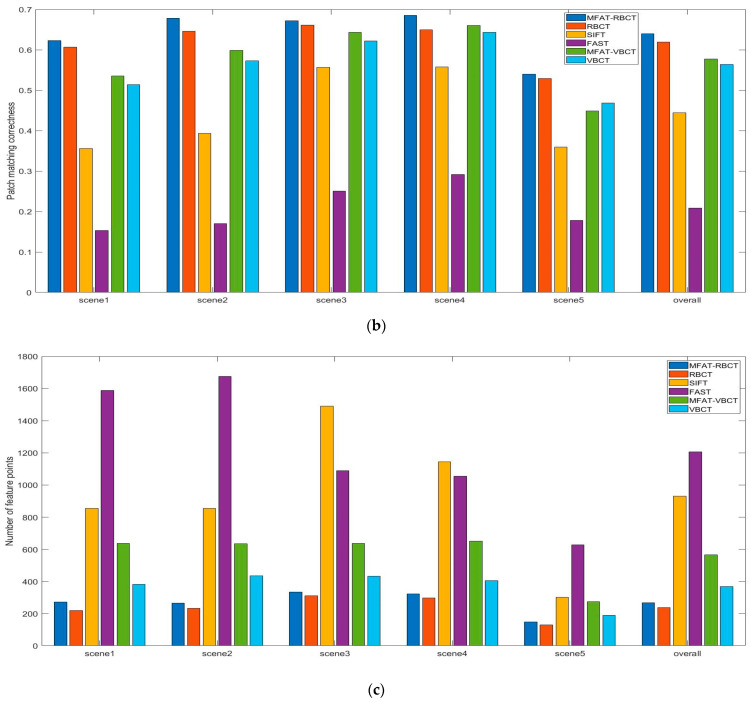
Indicator analysis: (**a**) comparison of repeatability; (**b**) comparison of patch matching correctness; (**c**) comparison of number of feature points.

As shown in Figure 13a, b, and c, respectively, this study calculated the repeatability, matching accuracy, and number of branch points for five data sets and their synthesis. In the first four data sets, there was a significant improvement in repeatability, matching accuracy, and the number of branch points compared to RBCT and VBCT. Data set clip5 showed similar results to RBCT and VBCT due to poor image quality indicators. The results showed that compared with RBCT, the overall matching accuracy improved by 3.23%, the number of branch point detections increased by 13.08%, and the repeatability improved by 4.91%, which meant that our method has achieved good performance on five data sets, proving its generalization and robustness.

## 5. Conclusions

The texture features in the human lumen environment are mainly vascular morphology, and feature point extraction in this environment is based on blood vessels. Branch points of blood vessels can still be detected in lumen blood vessels with a single texture feature and strong robustness even under rotation scaling and other conditions. This study proposed a vascular feature detection and matching method based on dual-branch weighted fusion vascular structure enhancement. A universal preprocessing framework and Gaussian weighted fusion of dual-branch Frangi measure and MFAT for single-pixel enhancement of the vascular structure was designed. In addition, this study utilized dual-circle detection and NMS to extract features and calculate the ZSSD algorithm for neighborhood blocks to achieve feature correlation. The experimental results showed that compared to RBCT, the overall matching accuracy improved by 3.23%, the number of branch point detections increased by 13.08%, and the repeatability improved by 4.91%. Through quantitative analysis of multiple indicators, it has been proven that the method proposed in this paper can better handle vascular structures with robustness and generalization, and is a feature detection and matching method suitable for various lumen environments and can provide more robust feature information for subsequent 3D reconstruction.

After conducting more thorough tracking of the internal cavity features, we are exploring the possibility of incorporating this method into the visual odometry section to obtain three-dimensional information about the human internal cavity. The 3D virtual image is superimposed in real time on the imaging display to achieve an augmented reality display effect of the 3D MIS screen from the doctor’s viewpoint. This provides feedback on the depth of the internal cavity and expands the doctor’s surgical field of view. It also helps to reduce surgical trauma, alleviate the patient’s pain, and improve the quality of the surgery. The technology seeks to promote its better application in the field of minimally invasive surgery and clinical trials.

## Figures and Tables

**Table 1:** The eigenvalues of the Hessian matrix.

*λ*_1_	*λ*_2_	Directional Feature
L	L	Background, no direction
N	N	Noise, directionless
L	H−	Tubular structure (bright)
L	H+	Tubular structure (dark)

**Table 2:** Maximum SSIM score for data set base images.

Data Set	Best Weight Parameter for SSIM	Best SSIM Score
clip1	3.9	0.07547
clip2	3.8	0.07186
clip3	2.1	0.13532
clip4	3.7	0.00204
clip5	3.6	0.04381

**Table 3:** Comparison of feature point extraction algorithms.

Algorithms	Data Set	Image I	Image J	Repeatability	Average Error	Variance
		Number of Branch Points				
AFAST	clip1	1368	1409	0.162913	2.047352	0.785429
	clip2	1493	1628	0.188212	2.037728	0.840030
	clip3	1023	882	0.281159	1.984439	0.678100
	clip4	801	1019	0.134831	2.174954	0.629708
	clip5	699	545	0.294372	1.922701	0.866902
RBCT	clip1	163	177	0.361963	1.866042	0.718423
	clip2	197	237	0.441624	1.614276	0.682030
	clip3	304	333	0.579710	1.752783	0.755851
	clip4	215	277	0.302326	1.745650	0.717040
	clip5	161	117	0.440367	1.661636	0.597105
OURS (MFAT- RBCT)	clip1	276	278	0.433460	1.770989	0.670291
	clip2	354	378	0.497175	1.571009	0.691472
	clip3	406	376	0.668033	1.744668	0.783226
	clip4	237	315	0.320675	1.724399	0.739681
	clip5	184	158	0.452055	1.504729	0.690412

## Data Availability

http://www.cse.usf.edu/~bingxiong/Vessel_Datasets.zip (accessed on 31 July 2014).
